# Radiotherapeutic Management of Synchronous Prostate and Rectal Cancers Using Proton Beam Therapy

**DOI:** 10.14338/IJPT-20-00087.1

**Published:** 2021-04-20

**Authors:** Jennifer S. Chiang, Nathan Y. Yu, Janina T. Sheedy, Robin E. Hayden, Pamela R. Lemish, Nina J. Karlin, Nitin Mishra, Terence T. Sio

**Affiliations:** 1 Department of Radiation Oncology, Mayo Clinic, Phoenix, AZ, USA; 2 Department of Hematology and Medical Oncology, Mayo Clinic, Phoenix, AZ, USA; 3 Department of Colon and Rectal Surgery, Mayo Clinic, Phoenix, AZ, USA

**Keywords:** synchronous cancers, rectal, prostate, proton beam therapy

## Abstract

Treatment of synchronous prostate and rectal cancers is a rare yet challenging problem with compounded toxicities. We report a case of a 65-year-old man who underwent proton beam therapy (PBT) with concurrent capecitabine and hormonal therapy for his synchronously found prostate (intermediate-risk) and rectal (cT2, N2b, stage IIIB) cancers; he also received low anterior resection. Before PBT, the patient experienced hematochezia. His baseline American Urological Association symptom score was a total of 0, and he was not sexually active. He completed PBT with grade 1 acute toxicities including fatigue, nausea, and increased urinary and bowel frequencies. He also developed mild anemia (10.7), which was resolved. Subsequent surgical pathology showed a pathologic complete response in his rectum. At follow-up of 2.5 years, he remained disease-free on surveillance imaging for both malignancies and reported increased bowel urgency and frequency, minimal urinary leakage when having urgency, and peripheral neuropathy. This case, along with a succinct literature review, demonstrates that PBT can be successful in the definitive treatment of synchronous prostate and rectal cancers with minimal toxicities. Further research is required to evaluate the efficacy and side effect profiles of PBT.

## Introduction

Prostate and colorectal cancers are common malignancies, and radiation therapy (RT) plays a significant therapeutic role in both cancers. They both have high incidences in males; however, cases of synchronous prostate and rectal cancers are rare [1, 2]. A recent study reported metachronous colorectal and prostate cancers found in 6 (0.16%) of 3722 patients [3]. Separately, if detected early, these cancers are often curable with manageable treatment-related side effects. Treatment options include radiation therapy and surgery for rectal cancer and external beam radiation therapy for localized prostate cancer [4, 5]. However, the optimal multimodality management including options for various RT strategies for patients with synchronous prostate and rectal cancers is much less known. In the treatment of prostate cancer, lower late gastrointestinal (GI) toxicity rates have been reported with delivery of dose-escalated RT using proton beam therapy (PBT) or intensity-modulated radiation therapy (IMRT) compared to 3D conformal radiation therapy (3DCRT) [6]. Postprostatectomy PBT has also been shown to minimize low-range bladder and rectal doses relative to IMRT [7]. In the treatment of rectal cancer, dose-volume histogram analyses reveal significantly reduced doses to organs at risk (OARs), including the small bowel, testes, and bladder, using protons versus RapidArc, IMRT, and 3DCRT [8]. Here, we describe a case wherein the patient was treated with PBT with concurrent chemotherapy and hormonal therapy. Clinical pearls, oncologic rationales, and RT-based principles are comprehensively discussed.

## Case Report

**Figure 1.**
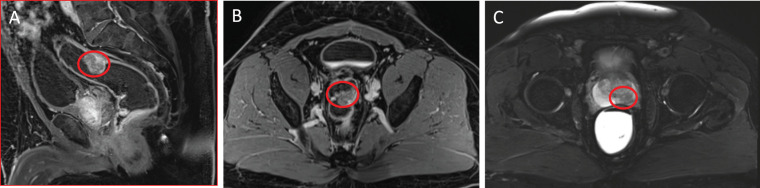
Sagittal (A) and axial (B) T1 MRI images with contrast showing a hypervascular mass involving the posterior rectum without evidence of extension into the perirectal fat (a T2 lesion). (C) Axial T2 MRI image showing a hypointense lesion in the posteromedial aspect of the peripheral zone of the mid prostate gland (organ confined and without extracapsular extension). Abbreviation: MRI, magnetic resonance imaging.

A 65-year-old man with synchronous prostate and rectal cancers was referred to us in August 2017. His baseline health was excellent. He experienced hematochezia, decreased stool caliber, and sensation of incomplete stool emptying at presentation. Laboratory findings included a carcinoembryonic antigen (CEA) level of 6.8 ng/mL. He underwent a colonoscopy that revealed a fungating, nonobstructive mass in the high rectum. Biopsy demonstrated an invasive moderately differentiated adenocarcinoma. Computed tomography abdomen/pelvis showed no evidence of metastases. Magnetic resonance imaging (MRI) demonstrated a clinical T2, N2b, stage IIIB, 2.8-cm rectal tumor (AJCC 8th edition [9]), along with a suspicious 1.6-cm lesion in the posteromedial aspect of the peripheral zone at the mid prostate that was clinically palpable. His total prostate-specific antigen (PSA) level was 14.1 ng/mL. A subsequent prostate biopsy confirmed adenocarcinoma, which was staged as Gleason 4+3, cT2a, N0, M0, intermediate-risk (**Figure 1**).

**Figure 2.**
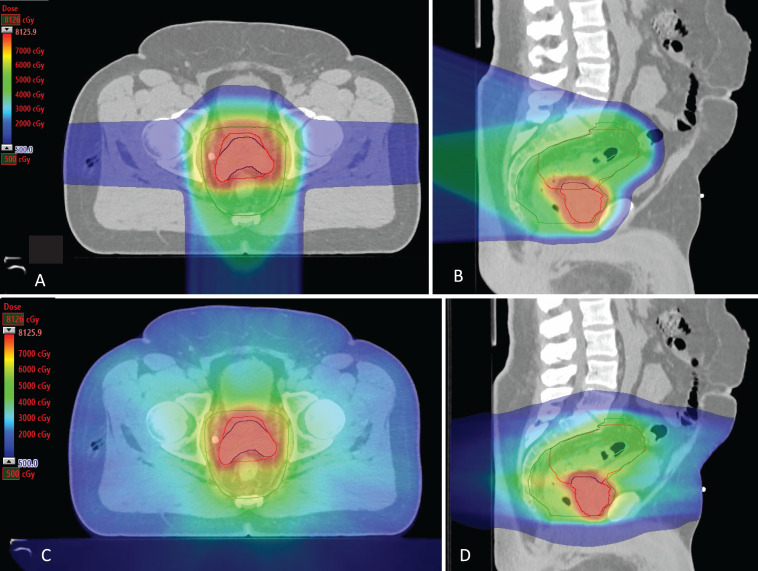
Axial (A) and sagittal (B) view of the proton beam therapy plan, encompassing irradiated volumes for both prostate and rectal cancers in a 65-year-old male patient. The GTV and CTV are shown here as CTV4500 (green, elective pelvic nodal volume), CTV5000 (rectal and prostate volumes, red), CTV7800 (sequential boost volume for prostate, blue), and GTV5000 (rectal and prostate volumes, cyan). Axial (C) and sagittal (D) images represent the same view levels of an IMRT comparison plan accordingly. Abbreviations: CTV, clinical target volume; GTV, gross target volume; IMRT, intensity-modulated radiation therapy.

Before treatment, his American Urological Association (AUA) symptom score was 0 of 35 at baseline. He was able to have erections but had not been sexually active. After a multidisciplinary discussion, the patient commenced PBT targeting both the rectum and the prostate, along with RT to pelvic nodes. For his rectal cancer, he received preoperative chemoradiation therapy with 50 GyE in 25 fractions (including the entire prostatic volume) and concurrent capecitabine. He received a boost of 78 GyE in 39 fractions to the prostate via a sequential boost plan (**Figure 2**). The initial rectal plus prostatic plan was given by 2 posterior beams (T270G150 and T270G185 beam angles), and the prostatic boost plan was given by opposed lateral beams. The patient was simulated in the supine position with standard pelvic immobilization and a full bladder. A rectal balloon was not used. Robust optimization was used and planned to the clinical target volume. Ultrasound-guided prostate marker seeds were used for daily image guidance. Dosimetric constraints used for the bladder (V80Gy ≤ 10%, V75Gy ≤ 15%, V65Gy ≤ 40%), rectum (D2cc < 81Gy, V70Gy ≤ 15%, V60Gy ≤ 30%), and small bowel (max point dose < 52 Gy, V50Gy < 2 cm^3^, V45Gy < 150 cm^3^) were met with the intensity-modulated proton therapy plan. A comparison plan is provided in **Table 1**. He completed the prescribed course of RT, concurrent chemotherapy, and 6 months of concurrent leuprolide therapy with no grade 3 or greater toxicities. In January 2018, MRI of the pelvis demonstrated significant decrease in size of the rectal mass. His total PSA level was 0.22 ng/mL. In February 2018, the patient underwent a robotic-assisted low anterior resection (LAR) with diverting loop ileostomy and bilateral ureteral stent placement. Final rectal pathology revealed a complete pathologic response including negative lymph nodes. The patient then completed 6 cycles of adjuvant XELOX chemotherapy. In August 2018, his AUA score remained at 0, and he reported no pain, urinary incontinence, dysuria, or hematuria. He had lost his erectile function.

**Table 1.** Proton versus IMRT dose to organs at risk for a patient with rectal and prostatic carcinomas.

**Dosimetric parameters**	**Protons (IMPT)**	**IMRT**
Bladder		
V80Gy (%)	0.0	0.3
V75Gy (%)	5.0	2.9
V70Gy (%)	7.6	5.1
V65Gy (%)	9.8	9.3
V40Gy (cm^3^)	67	109
Rectum		
V75Gy (%)	7.7	1.4
V70Gy (%)	13.0	5.3
V60Gy (%)	21.9	24.0
V40Gy (cm^3^)	52	99
Small bowel		
Max dose (Gy)	50.9	52.8
V30Gy (cm^3^)	5.2	10.8

**Abbreviations:** IMPT, intensity-modulated proton therapy; IMRT, intensity-modulated radiation therapy.

The patient completed ileostomy closure in February 2019, and his PSA level was 0.32 ng/mL at that time. Colonoscopy findings in May 2019 were normal. He reported no dysuria or hematuria. He reported regular bowel movements and formed stools, with slightly increased bowel urgency and frequency since ileostomy closure. In February 2020, he reported occasional minimal urinary leakage. His AUA score remained stable at 15 points. At his latest follow-up, in December 2020 (37 months since end of PBT), he reported only grade 1 chronic side effects. Regarding GI symptoms, he reported tenesmus leading to 2 to 5 bowel movements per day with formed stool. He reported no fecal incontinence or hematochezia. For genitourinary symptoms, his total AUA score was 10 (moderate), and he reported increased urgency, frequency, and difficulty emptying the bladder. He reported no hematuria or urinary incontinence. He continued to have no erectile function. His Eastern Cooperative Oncology Group (0000) score was 0. His PSA level was 0.35 ng/mL, and his CEA level was 2.5 ng/mL. Surveillance imaging showed no evidence of new metastatic disease from either cancer.

## Discussion

In our case study with follow-up of 3 years, we demonstrated that PBT for both prostate and rectal cancer was safe and efficacious. To our knowledge, this is the first known report of PBT use in the treatment of synchronous rectal and prostate cancers.

A meta-analysis found that use of PBT or IMRT was associated with significant decreases in the reported rate of severe GI toxicity as compared to 3D RT [6]. Postprostatectomy PBT has also been shown to minimize low-range bladder and rectal doses relative to IMRT, while further studies are needed to determine whether dosimetric differences will confer clinically meaningful differences in long-term outcomes [7]. In the treatment of rectal cancer, dose-volume histogram analyses reveal significantly reduced doses to OARs, including the small bowel, testes, and bladder, using protons versus RapidArc, IMRT, and including 3DCRT [8]. In several dosimetric analyses, PBT has also been shown to reduce V5Gy, V10Gy, V15Gy, and V20Gy to bone marrow, V10Gy and V20Gy to small bowel, and V40Gy to the bladder [8, 10–13]. Lower V10 to the pelvic bone marrow has been associated with lower rates of significant cytopenia for patients being treated with pelvic radiation [14]. Thus, bone marrow sparing may particularly benefit patients undergoing myelosuppressive chemotherapy [15]. Although there is no conclusive evidence that PBT is superior to IMRT for either prostate cancer or rectal cancer individually, this case report highlights the safety and efficacy of synchronous treatment with PBT. Further studies are needed to determine whether the dosimetric advantages of PBT in comparison to IMRT are clinically relevant.

Research on curative treatment options for *both* synchronous prostate and rectal cancers is limited (**Table 2**). In 2 case series, radical retropubic prostatectomy was safely performed with rectal resection [16, 17]. However, risk of urinary incontinence or fistula formation between the bowel and bladder due to overlying anastomoses is a major concern with combined proctectomy and LAR of the rectum [16, 17]. In the first study, 1 patient experienced an early postoperative small-bowel obstruction and ischemic colostomy, and another patient developed strictures of the rectal anastomosis and bladder anastomosis [16]. In the second study, 2 patients remained asymptomatic after 20 and 24 months of follow-up after surgery, while another developed recurrence 47 months after surgery [17]. In patients with synchronous rectal and prostate cancers, treatment of both tumors with RT, 3DCRT, and IMRT, and concomitant 5-fluorouracil has been documented [18–20]. However, the delivered doses were less than the recommended 78 Gy to the prostate in the modern era, and 1 patient treated with hypofractionated RT to the prostate experienced grade 2 enteritis [19]. Qiu et al [21] published a case series where RT was delivered by external beam RT and a brachytherapy boost followed by anterior resection in 4 patients, one of whom experienced a grade 3 GI toxicity (precluding receipt of adjuvant chemotherapy and ileostomy reversal). In a 5-patient retrospective review, postoperative morbidity included wound infection (2 patients), intra-abdominal collection requiring drainage (1 patient), and common peroneal neuropathy (1 patient) [22]. In a more recent retrospective review of 10 patients who were treated with IMRT or 3DCRT, chemotherapy, and surgery, 9 patients remained alive, either with no evidence of disease (5 patients) or living with metastatic disease (4 patients) [23]. Significant late toxicities were found.

**Table 2.** RT for synchronous rectal and prostate cancers—literature review.

	**Siu et al [18] (2001)**	**Colonias et al [19] (2005)**	**Watkins et al [20] (2012)**	**Qiu et al [21] (2012)**	**Kavanagh et al [22] (2012)**	**Lavan et al [23] (2016)**	**Present study**
No. of pts	2	1	1	4	4	10	1
Types of RT	3DCRT	IMRT	IMRT	EBRT + ILRT	3DCRT	IMRT or 3DCRT	PBT
Radiation dose/ No. of fractions	50.4 Gy + 60.4 Gy (rectal) 45 Gy + 20 Gy (prostate)	50.4 Gy/28 fractions (rectal) 70 Gy/28 fractions (prostate)	50.4 Gy (rectal) 79.2 Gy (prostate)	45–50.4 Gy (EBRT) 80–90 Gy (ILRT)	45 Gy (rectal) 74 Gy (prostate)	45–50.4 Gy (rectal) 70–74 Gy (prostate)	50 GyE/25 fractions (rectal) 78 GyE/39 fractions (prostate)
No. of pts treated with concurrent chemotherapy	1	1	1	3	4	9	1
No. pts treated with ADT	-	1	-	1	1	4	1
Surgery	Inoperable	Proctosigmoidectomy, colonic J-pouch and diverting loop ileostomy	LAR	LAR	2 pelvic exenterations	1 pelvic exenteration, 1 APR, 7 LARs	LAR
No. of pts treated with adjuvant chemotherapy	-	1	-	3	-	8	1
No. of pts with NED	2	1	1	3	2	5	1
Follow-up	1–2 y	14 mo postsurgery	1 y from diagnosis	38–62 mo postdiagnosis	3–120 mo	Median 2.2 y	2.5 y
Toxicities	N/A	Grade 2 enteritis during chemoradiation; no significant GI or GU toxicity at last follow-up	N/A	3 grade 1–2, 1 grade-3 GI toxicity	3 grade 1	1 grade-3 proctitis, 1 grade-3 anastomotic stricture	Grade 1

**Abbreviations:** pts, patients; RT, radiation therapy; 3DCRT, 3D conformal radiation therapy; IMRT, intensity-modulated radiation therapy; EBRT, external beam radiation therapy; ILRT, intraluminal radiation therapy; PBT, proton beam therapy; ADT, androgen deprivation therapy; LAR, low anterior resection; APR, abdominoperineal resection; NED, no evidence of disease; GI, gastrointestinal; GU, genitourinary; N/A, not available.

Studies have demonstrated the potential dosimetric benefits of PBT in the treatment of rectal and prostate cancers separately, including significant sparing of bone marrow for future systemic therapies [24, 25]. Reducing irradiated bowel volume may also allow for dose escalation in future studies, which was a limiting factor in a prior phase II trial (RTOG 0247) [26]. Proton beam therapy may play a significant role in limiting long-term toxicities for patients with synchronous pelvic malignancies.

In summary, we demonstrated that PBT with concurrent chemotherapy and hormonal therapy can be successful for synchronous prostate and rectal cancers. The superior dosimetric advantages and lower delivered dose outside of the target tissue afforded by PBT may have contributed to the therapeutic success for this case with minimal bowel and urinary toxicities and myelosuppression in the long-term. Further studies with more patients are needed to evaluate the efficacy of PBT in the treatment of synchronous pelvic cancers in the future.

## References

[1] Lee TK, Barringer M, Myers RT, Sterchi JM (1982). Multiple primary carcinomas of the colon and associated extracolonic primary malignant tumors. *Ann Surg*.

[2] Weir JA (1975). Colorectal cancer: metachronous and other associated neoplasms. *Dis Colon Rectum*.

[3] Chiang JM, Yeh CY, Changehien CR, Chen JS, Tang R, Tsai WS, Fan CW (2004). Clinical features of second other-site primary cancers among sporadic colorectal cancer patients—a hospital-based study of 3,722 cases. *Hepatogastroenterology*.

[4] Minsky BD (2019). Emerging trends in the treatment of rectal cancer. *Acta Oncol*.

[5] Brawley S, Mohan R, Nein CD (2018). Localized prostate cancer: treatment options. *Am Fam Physician*.

[6] Ohri N, Dicker AP, Showalter TN (2012). Late toxicity rates following definitive radiotherapy for prostate cancer. *Can J Urol*.

[7] Santos PMG, Barsky AR, Hwang WT, Deville C, Wang X, Both S, Bekelman JE, Christodouleas JP, Vapiwala N (2019). Comparative toxicity outcomes of proton-beam therapy versus intensity-modulated radiotherapy for prostate cancer in the postoperative setting. *Cancer*.

[8] Wolff HA, Wagner DM, Conradi LC, Hennies S, Ghadimi M, Hess CF, Christiansen H (2012). Irradiation with protons for the individualized treatment of patients with locally advanced rectal cancer: a planning study with clinical implications. *Radiother Oncol*.

[9] Amin MB, Edge S, Greene F, Byrd DR, Brookland RK, Washington MK, Gershenwald JE, Compton CC, Hess KR, Sullivan DC, Jessup JM, Brierley JD, Gaspar LE, Schilsky RL, Balch CM, Winchester DP, Asare EA, Madera M, Gress DM, Meyer LR (2017). American Joint Committee on Cancer *AJCC Cancer Staging Manual* 8th ed.

[10] Colaco RJ, Nichols RC, Huh S, Getman N, Ho MW, Li Z, Morris CG, Mendenhall WM, Mendenhall NP, Hoppe BS (2014). Protons offer reduced bone marrow, small bowel, and urinary bladder exposure for patients receiving neoadjuvant radiotherapy for resectable rectal cancer. *J Gastrointest Oncol*.

[11] Tatsuzaki H, Urie MM, Willett CG (1992). 3-D comparative study of proton vs. x-ray radiation therapy for rectal cancer. *Int J Radiat Oncol Biol Phys*.

[12] Palmer M, Mok H, Ciura K, Georges R, Nguyen B, Crawford C, Beddar S, Zhu R, Crane C, Das P (2012). Dose reduction to small bowel and other relevant structures in rectal carcinoma with proton therapy. *Int J Radiat Oncol Biol Phys*.

[13] Blanco Kiely JP, White BM (2016). Retracted: robust proton pencil beam scanning treatment planning for rectal cancer radiation therapy. *Int J Radiat Oncol Biol Phys*.

[14] Mell LK, Schomas DA, Salama JK, Devisetty K, Aydogan B, Miller RC, Jani AB, Kindler HL, Mundt AJ, Roeske JC, Chmura SJ (2008). Association between bone marrow dosimetric parameters and acute hematologic toxicity in anal cancer patients treated with concurrent chemotherapy and intensity-modulated radiotherapy. *Int J Radiat Oncol Biol Phys*.

[15] Alex S, Brooks ED, Holliday EB (2019). Proton therapy for colorectal cancer. *Appl Radiat Oncol*.

[16] Klee LW, Grmoljez P (1999). Combined radical retropubic prostatectomy and rectal resection. *Urology*.

[17] Lin C, Jin K, Hua H, Lin J, Zheng S, Teng L (2011). Synchronous primary carcinomas of the rectum and prostate: report of three cases. *Oncol Lett*.

[18] Siu W, Kapp DS, Wren SM, King C, Terris MK (2001). External beam radiotherapy for synchronous rectal and prostatic tumors. *Urology*.

[19] Colonias A, Farinash L, Miller L, Jones S, Medich DS, Greenberg L, Miller R, Parda DS (2005). Multidisciplinary treatment of synchronous primary rectal and prostate cancers. *Nat Clin Pract Oncol*.

[20] Watkins JM, Wos E, Berglund D, Colby K, Dufan TA, Koleilat N (2012). Synchronous presentation of advanced rectal and intermediate-risk prostate cancers: a multidisciplinary approach. *Commun Oncol*.

[21] Qiu H, Herman JM, Ahuja N, DeWeese TL, Song DY (2012). Neoadjuvant chemoradiation followed by interstitial prostate brachytherapy for synchronous prostate and rectal cancer. *Pract Radiat Oncol*.

[22] Kavanagh DO, Quinlan DM, Armstrong JG, Hyland JM, O'Connell PR, Winter DC (2012). Management of synchronous rectal and prostate cancer. *Int J Colorectal Dis*.

[23] Lavan NA, Kavanagh DO, Martin J, Small C, Joyce MR, Faul CM, Kelly PJ, O'Riordain M, Gillham CM, Armstrong JG, Salib O, McNamara DA, McVey G, O'Neill BD (2016). The curative management of synchronous rectal and prostate cancer. *Br J Radiol*.

[24] Trofimov A, Nguyen PL, Coen JJ, Doppke KP, Schneider RJ, Adams JA, Bortfeld TR, Zietman AL, Delaney TF, Shipley WU (2007). Radiotherapy treatment of early-stage prostate cancer with IMRT and protons: a treatment planning comparison. *Int J Radiat Oncol Biol Phys*.

[25] Sauer R, Becker H, Hohenberger W, Rodel C, Wittekind C, Fietkau R, Martus P, Tschmelitsch J, Hager E, Hess CF, Karstens JH, Liersch T, Schmidberger H, Raab R (2004). Preoperative versus postoperative chemoradiotherapy for rectal cancer. *New Engl J Med*.

[26] Wong SJ, Winter K, Meropol NJ, Anne PR, Kachnic L, Rashid A, Watson JC, Mitchell E, Pollock J, Lee RJ, Haddock M, Erickson BA, Willett CG (2012). Radiation Therapy Oncology Group 0247: a randomized Phase II study of neoadjuvant capecitabine and irinotecan or capecitabine and oxaliplatin with concurrent radiotherapy for patients with locally advanced rectal cancer. *Int J Radiat Oncol Biol Phys*.

